# The active metabolite of leflunomide, A77 1726, attenuates inflammatory arthritis in mice with spontaneous arthritis via induction of heme oxygenase-1

**DOI:** 10.1186/s12967-017-1131-x

**Published:** 2017-02-13

**Authors:** Su-Jin Moon, Eun-Kyung Kim, Joo Yeon Jhun, Hee Jin Lee, Weon Sun Lee, Sang-Hi Park, Mi-La Cho, Jun-Ki Min

**Affiliations:** 1 Division of Rheumatology, Department of Internal Medicine, College of Medicine, The Catholic University of Korea, Seoul, South Korea; 2 The Rheumatism Research Center, Catholic Research Institute of Medical Science, The Catholic University of Korea, Seoul, South Korea; 3 Clinical Medicine Research Institute of Bucheon St. Mary’s Hospital, The Catholic University of Korea, Bucheon-si, Gyeonggi-do South Korea

**Keywords:** Rheumatoid arthritis, Leflunomide, Heme oxygenase-1

## Abstract

**Background:**

Leflunomide is a low-molecular-weight compound that is widely used in the treatment of rheumatoid arthritis. Although leflunomide is thought to act through the inhibition of the de novo pyrimidine synthesis, the molecular mechanism of the drug remains largely unknown. We investigated the antiarthritis effects and mechanisms of action of the active metabolite of leflunomide, A77 1726, in interleukin-1 receptor antagonist-knockout (IL-1Ra-KO) mice.

**Methods:**

14- to 15-week-old male IL-1Ra-KO mice were treated with 10 or 30 mg/kg A77 1726 via intraperitoneal injection three times per week for 6 weeks. The effects of A77 1726 on arthritis severities were assessed by clinical scoring and histological analysis. The serum concentrations of IL-1β, tumor necrosis factor-α (TNF-α), and malondialdehyde were measured by enzyme-linked immunosorbent assay. Histologic analysis of the joints was performed using Safranin O, and immunohistochemical staining. The frequencies of interleukin-17-producing CD4^+^ T (Th17) cells were analyzed by flow cytometry. Heme oxygenase-1 (HO-1) expression in splenic CD4^+^ T cells isolated from A77 1726-treated arthritis mice were assessed by western blotting.

**Results:**

A77 1726 treatment induced heme oxygenase-1 (HO-1) in Jurkat cells and primary mouse T cells. Interestingly, A77 1726 inhibited Th17 cell differentiation. In vivo, A77 1726 reduced the clinical arthritis severity of histological inflammation and cartilage destruction. The joints isolated from A77 1726-treated mice showed decreased expression of inducible nitric oxide synthase, nitrotyrosine, TNF-α, and IL-1β. The serum levels of TNF-α, IL-1β, and malondialdehyde were also decreased in A77 1726-treated mice. Whereas the number of Th17 cells in spleens was decreased in A77 1726-treated arthritis mice, a significant increase in the number of Treg cells in spleens was observed. Interestingly, HO-1 expression was significantly higher in splenic CD4^+^ T cells isolated from A77 1726-treated mice compared with those from vehicle-treated mice, whereas HO-1 expression of splenic non-CD4^+^ T cells did not differ between groups.

**Conclusion:**

The inhibitory effects of A77 1726 on joint inflammation and oxidative stress in autoimmune arthritis may be associated with HO-1 induction in CD4^+^ T cells.

## Background

Rheumatoid arthritis (RA) is a debilitating, chronic autoimmune polyarthritis, characterized by synovial hypertrophy and inflammatory cell infiltration in the affected joints. Although the pathogenesis of RA remains elusive, it is known to involve many cell types, including CD4^+^ T cells and B cells, in the inflamed hypertrophic synovium, called “pannus”. These cells play pathological roles in the development of RA by producing cytokines that perpetuate rheumatoid inflammation [1]. Therefore, the major targets of RA treatment are the proinflammatory immune cells, especially CD4^+^ T cells, which are a pivotal player in the development and progression of RA, and their production of inflammatory cytokines, such as tumor necrosis factor-α (TNF- α), interleukin 1β (IL-1β), and IL-17. Among the T cell subsets, a distinct lineage of IL-17-producing effector T helper cells, called Th17 cells, have been identified to be involved in the pathogenesis of RA [2]. On the opposite side of Th17 cells, there are Foxp3-expressing regulatory T (Treg) cells that have a pivotal role in suppressing immune responses [3]. Accumulating scientific evidences have revealed that many autoimmune diseases including RA result from an imbalance between Treg cells and Th17 cells [4–6]. The potential role of Treg cells as a treatment target for RA has attracted increasing interest [7]. Furthermore, the plasticity between Treg and Th17 cells indicates that Th17/Treg imbalance plays a critical role during the development and progression of RA [8, 9]. Thus, the reciprocal regulation of Th17/Treg subsets can be a novel therapeutic strategy for RA.

Although the pathogenesis of RA is not understood clearly, altered oxidative stress is considered to play a critical role during the development and perpetuation of the disease [10, 11]. Over the course of evolution, defensive mechanisms against oxidative stress have been developed. One major mechanism in the cellular defense against oxidative stress is activation of the nuclear-related factor 2 (Nrf2), a key transcription factor that contributes to the maintenance of cellular redox homeostasis [12]. One of the major stress-responsive players regulated by Nrf2 is the antioxidant enzyme heme oxygenase-1 (HO-1). Nrf2 signaling has received recent attention as a participant in the pathogenesis of RA. A deficiency in the Nrf2 pathway in an animal model of RA was associated with aggravated arthritis severity and osteoporosis, and may indicate the potential protective role of functional Nrf2–HO-1 signaling in RA [13]. It has also been reported that impairment in the antioxidant systems leads to increased oxidative stress in RA [14].

Leflunomide is an oral drug that inhibits de novo pyrimidine synthesis by inhibiting dihydroorotate dehydrogenase and was originally developed for the synthesis of agricultural pesticides [15]. Leflunomide exerts immunoregulatory properties and is useful in RA treatment, and has been introduced as a synthetic disease-modifying antirheumatic drug (DMARD) [16]. The immunomodulatory action of leflunomide is mediated primarily through the effect of its water-soluble metabolite, termed A77 1726. Despite the clinical usefulness of the drug, the underlying mechanisms of the drug remain elusive. The immunosuppressive activity has been shown to be independent of pyrimidine synthesis inhibition [17, 18].

Although many DMARDs are believed to affect oxidative stress in RA, there is insufficient research data to confirm this circumstantial assumption. Thus, in the present study, we explored the in vivo effects of the active metabolite of leflunomide, A77 1726, in an animal model of RA. We used IL-1 receptor antagonist-knockout (IL-1Ra-KO) mice to investigate the underlying mechanisms of action of A77 1726 and aspects of oxidative stress production.

## Methods

### Mice

IL-1Ra-KO mice with the BALB/c background were kindly provided by Professor Yoichiro Iwakura (University of Tokyo, Japan) and were maintained under specific pathogen-free conditions at the Institute of Medical Science, Catholic University of Korea, and fed standard mouse chow (Ralston Purina, St Louis, MO, USA) and water ad libitum. All experimental procedures were examined and approved by the Animal Research Ethics Committee of the Catholic University of Korea, which conformed to all National Institutes of Health of the USA guidelines. Mice were euthanized at the end of a study for the purpose of sample collection and histologic examination by CO_2_ chamber. The experimental protocol was approved, and all animals were treated and sacrificed in accordance with the guidelines of the Catholic university of Korea on Use and Care of Animals.

### Treatment with A77 1726

To assess the therapeutic effect of A77 1726 on symptom severity in the IL-1Ra-KO mouse model, 14–15-week-old male IL-1Ra-KO mice were treated with 10 or 30 mg/kg A77 1726 (Santa Cruz Biotechnology, Santa Cruz, CA, USA) dissolved in dimethyl sulfoxide (DMSO) or with vehicle alone, via intraperitoneal injection three times per week for 6 weeks. The clinical signs of arthritis in IL-1Ra-KO mice were monitored two times a week by inspection and were quantified using an arthritis score, as reported previously [19]. The final arthritis score was calculated as the sum of scores from all four legs, which were assessed by three independent observers with no knowledge of the experimental groups.

### Tissue scoring

The H&E stained sections were scored for inflammation and bone erosion. Inflammation was scored according to the following criteria [20] : 0 = no inflammation, 1 = slight thickening of the lining layer or some infiltrating cells in the underlying layer, 2 = slight thickening of the lining layer plus some infiltrating cells in the underlying layer, 3 = thickening of the lining layer, an influx of cells in the underlying layer and the presence of cells in the synovial space, and 4 = synovium highly infiltrated with many inflammatory cells. Cartilage damage was determined using safranin-O staining, and the extent of cartilage damage was scored according to the following criteria: 0 = no destruction, 1 = minimal erosion limited to single spots, 2 = slight to moderate erosion in a limited area, 3 = more extensive erosion, and 4 = general destruction [20]. The extent of bone erosion was expressed using a scoring system from 0 to 5 (0 = no erosion, 1 = small areas of resorption not readily apparent at low magnification, in the trabecular or cortical bone, 2 = more numerous areas of resorption, not readily apparent at low magnification, in the trabecular or cortical bone, 3 = obvious resorption of the trabecular and cortical bone, without full-thickness defects in the cortex, loss of some trabeculae, lesions apparent at low magnification, 4 = full-thickness defects in the cortical bone and marked trabecular bone loss, without distortion of the profile of the remaining cortical surface, and 5 = full-thickness defects in the cortical bone and marked trabecular bone loss, with distortion of the profile of the remaining cortical surface), as previously described [21].

### T cell isolation from each group of mice

On day 42 after the first A77 1726 injection, the spleen was removed from each mouse in the different groups. The spleen tissue was minced. Splenic red blood cells were removed with an ACK lysis buffer (2.06% Tris [pH 7.65], 0.83% NH4Cl). The cell suspension was passed through a 40 μm strainer (BD Falcon, Bedford, MA, USA) and resuspended in 5% fetal bovine serum (Gibco, Grand Island, NY, USA) containing RPMI 1640 (Gibco) medium. Spleen cells were washed with 0.5% bovine serum albumin (BSA, Sigma, St. Louis, MO, USA) and 5 mM ethylenediaminetetraacetic acid (EDTA, Sigma) containing phosphate-buffered saline (PBS) buffer (pH 7.2). After centrifugation at 1300 rpm and 4 °C, the cells were incubated with CD4-coated magnetic beads (Miltenyi Biotec, Bergisch Gladbach, Germany) and isolated on MACS separation columns (Miltenyi Biotec). Positively selected CD4^+^ T cells were collected for western blotting and real-time polymerase chain reaction (PCR).

### Histology and immunohistochemical analysis

Mouse joint tissue was fixed in 4% paraformaldehyde, decalcified in EDTA bone decalcifier, and embedded in paraffin. Tissue sections (7 μm) were prepared and stained with hematoxylin and eosin (H&E), and Safranin O to enable evaluation of proteoglycan content. Confocal microscopy was used to detect immunostaining for IL-17-producing CD4^+^ T (Th17) and CD4^+^ CD25^+^ Foxp3^+^ regulatory T (Treg) cells, as previously described [22]. Slides for immunohistochemistry were deparaffinized and rehydrated using a graded ethanol series. The sections were depleted of endogenous peroxidase activity by adding methanolic H_2_O_2_ and then blocked with normal goat serum for 30 min. The samples were incubated overnight at 4 °C with antibodies to IL-1β at a dilution of 1:50 (Santa Cruz Biotechnology), inducible nitric oxide synthase (iNOS) at 1:100 (Abcam), nitrotyrosine at 1:100 (Santa Cruz Biotechnology), and TNF-α. The samples were then incubated with the relevant secondary antibody, biotinylated anti-mouse IgG or rabbit IgG, for 20 min, conjugated to a streptavidin peroxidase complex (Vector Laboratories) for 1 h, and finally with 3,3′-diaminobenzidine (Dako). To detect HO-1 expressing splenocytes, immunohistochemistry was performed using the Vectastain ABC kit. Tissues were first incubated with primary anti-HO-1 antibodies overnight at 4  °C. The primary antibody was detected with a biotinylated secondary antibody followed by incubation with a streptavidin-peroxidase complex for 1  h. DAB chromogen was added to obtain colored product. The sections were counterstained with Mayer’s hematoxylin and photographed using an Olympus photomicroscope (Olympus, Tokyo, Japan).

### Confocal and immunofluorescence microscopy

Tissues were obtained 43 days after first injection of A77 1726, snap-frozen in liquid nitrogen, and stored at −80 °C. Tissue cryosections (7 μm thick) were fixed and permed in acetone and stained for Tregs using allophycocyanin (APC)-labeled anti-CD25 (Biolegend), phycoerythrin (PE)-labeled anti-FoxP3 (eBioscience), and Alexa488-conjugated anti-CD4 (Biolegend). To stain Th17 cells, PE-labeled anti-IL-17 (eBioscience), Alexa Fluor ® 488-conjugated anti-CD4 (BioLegend), PE-labeled anti-STAT3 phosphorylation at tyrosine 705 (pSTAT3Tyr705) or at serine 727 (pSTAT3Ser727) (both from BD Biosciences) were used. To detect p-STAT5, the sections were incubated overnight at 4 °C with the primary antibody (Cell Signaling), and were followed by staining with PE-conjugated rabbit secondary antibody (Southern Biotech). After an overnight incubation at 4 °C, the stained sections were visualized by confocal microscopy (LSM 510 Meta; Zeiss, Göttingen, Germany). Jurkat cells were cultured at a density of 5 × 10^5^/ml in six-well plates, exposed to A77 1726 or vehicle (DMSO) for 24 or 48 h, harvested, and fixed at room temperature with 2% paraformaldehyde in PBS. The cells were washed three times with PBS for 5 min and permeabilized with 0.1% Triton X-100 in PBS. Nonspecific antibody binding was blocked by incubation with 5% normal goat serum in PBS for 1 h at room temperature. The cells were then incubated overnight at 4 °C with a rabbit polyclonal anti-Nrf2 antibody (1:100 dilution; Abcam) or anti-HO-1 antibody (1:250 dilution, Novus Biologicals, Littleton, CO, USA) and washed three times with PBS. Next, a secondary antibody (FITC-labeled goat anti-rabbit IgG, 1:200 dilution; Santa Cruz Biotechnology) was applied, and the cells were incubated in a dark chamber for 45 min, and then counterstained with DAPI for 5 min. The cells were washed with PBS, and antifade mounting medium (Gel-Mount; BioMeda) and a coverslip were applied. Staining was evaluated using a fluorescence microscope (BX50; Olympus).

### Murine T cell isolation and differentiation

The C57BL/6 (B6) mouse spleens were collected for cell preparation and washed twice with PBS. The spleens were minced, and the red blood cells were lysed with 0.83% ammonium chloride. The cells were filtered through a cell strainer and centrifuged at 1300 revolutions per minute for 5 min at 4 °C. To purify splenic CD4^+^ T cells, the splenocytes were incubated with CD4-coated magnetic beads and isolated using MACS separation columns (Miltenyi Biotec). Th17 cells were polarized with plate-bound anti-CD3 (0.5 μg/ml), soluble anti-CD28 (1 μg/ml), anti–interferon-γ (anti-IFNγ; 2 μg/ml), anti-IL-4 (2 μg/ml), IL-6 (20 ng/ml), and transforming growth factor β (TGF β; 2 ng/ml) for 72 h.

### Intracellular staining and flow cytometry

The following antibodies were used for intracellular staining of cells: PerCP-Cy5.5-conjugated anti-CD4, APC-conjugated anti-CD25, FITC-conjugated anti-IL-17A, and PE-conjugated anti-FoxP3 (all from eBioscience).

### Enzyme-linked immunosorbent assay (ELISA)

Sandwich ELISA kits were used to measure the amounts of IL-1β (R&D Systems, Minneapolis, MN, USA), TNF-α (R&D Systems), and malondialdehyde (MDA) (MyBioSource, Inc., San Diego, CA, USA) in sera obtained from the mice.

### HO-1 Real-time PCR and Western blot analysis

RNA was collected with RNeasy plus Mini Kit (QIAGEN, Valencia, CA, USA) from the cells purified CD4^±^ splencyte. Treatment with deoxyribonuclease 1 (QIAGEN) eliminated genomic DNA, and 1ug purified RNA was reverse transcribed into first-strand complementary DNA using an Quantitect reverse transcription kit (QIAGEN, Valencia, CA, USA), which includes a genomic DNA elimination step. Real-time polymerase chain reaction (RT-PCR) amplification and relative quantification of heme oxygenase 1 (Hmox1) was performed using TaqMan gene expression assays (Table 1) (Applied Biosystems, Foster City, CA, USA) on a lightcycler 480 PCR system (Roche, Mannheim, Germany). All assays used similar amplification efficiency, and a delta cycle threshold experimental design was used for relative quantification. The reactions were performed in triplicates in a 20 UL volume using TaqMan probe Master Mix (Roche), and 20 ng complementary DNA was used in each reaction. Glyceraldehyde 3-phosphate dehydrogenase served as an endogenous control. Results were analyzed using lightcycler 480 instrument software 1.2 (Roche, Mannheim, Germany). Protein concentration was determined using the Bradford method (Bio-Rad, Hercules CA, USA). Protein samples were separated using 10% sodium dodecyl sulfate–polyacrylamide gel electrophoresis and transferred to a nitrocellulose membrane (Amersham Pharmacia Biotech, NJ, USA). The membrane was preincubated with 5% skim milk in Tris-buffered saline (TBS) for 2 h at room temperature. Primary antibodies against HO-1 (Novus Biologicals), and β-actin (Cell Signaling Technology, Beverly, MA, USA) were diluted 1:1000 in 5% BSA-TBS with 0.1% Tween 20 (TBST) added, and the samples were incubated overnight at 4 °C. The samples were washed four times with TBST, horseradish peroxidase-conjugated secondary antibodies were added, and the samples were incubated for 1 h at room temperature. The samples were washed in TBST, and the hybridized bands were detected with an ECL detection kit (Pierce) and analyzed using a ChemiDoc XRS system with image Lab software (Bio-Rad Laboratories).

**Table 1:** Gene expression assays used for real-time polymerase chain reaction for mouse splenocyte

Gene	Abbreviation	Reference sequence	Assay number
Heme oxygenase 1	Hmox1	NM_010442.2	Mm00516005_m1
Glyceraldehyde-3-phosphate dehydrogenase	Gapdh	NM_001289726.1	Mm99999915_g1

## Statistical analysis

Statistical analysis was performed using IBM SPSS Statistics 20 for Windows (IBM Corp., Armonk, NY, USA). One-way analysis of variance followed by the Bonferroni post hoc test was used to compare differences between three or more groups. Comparison of numerical data between two groups was performed using the Mann–Whitney *U* test, and *P* values <0.05 were considered significant. The data are presented as the mean ± standard deviation (SD).

## Results

### A77 1726 induces Nrf2-HO-1 axis and inhibited Th17 differentiation in a dose-dependent manner in vitro

**Fig. 1:**
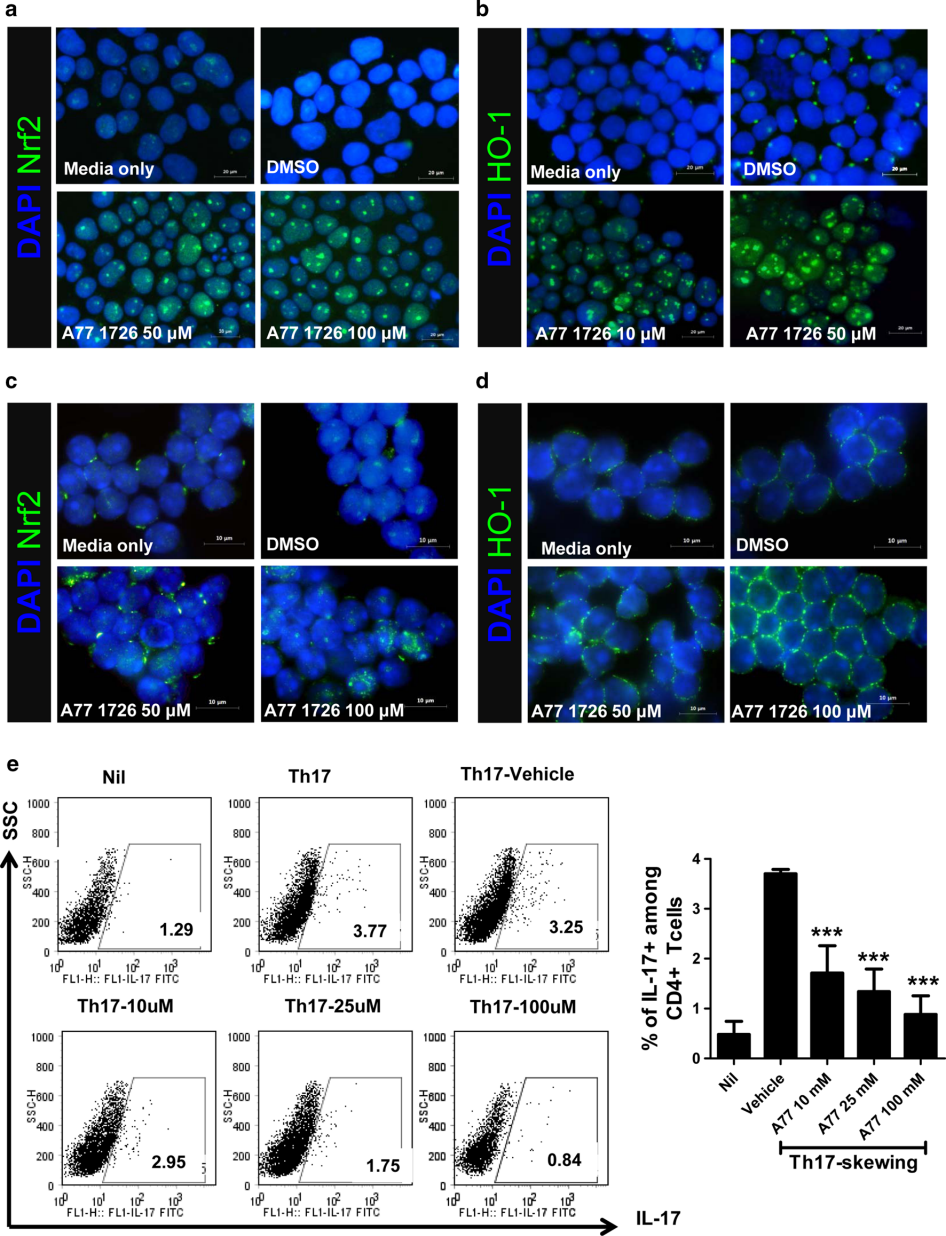
Activation of Nrf-HO-1 in Jurkat and murine CD4^+^ T cells upon exposure to A77 1726. The Nrf2 and HO-1 activity induced in Jurkat cells (**a**, **b**) and mouse T cells (**c**, **d**) in A77 1726-treated cells compared with vehicle (DMSO)-treated cells was determined by immunofluorescence microscopy. The cells were cultured for 48 h in the presence or absence of A77 1726 at concentrations ranging from 10 to 100 μM. **e** Splenic CD4^+^ T cells from C57BL/6 mice were cultured under Th17 cells-polarizing conditions in the presence or absence of A77 1726. Three days later, the cells were stained with antibodies against CD4 and IL-17 cells. A plot from 1 representative experiment shows the frequencies of IL-17^+^ cells among CD4^+^ T cells (*left*). Mean ± SD values are presented in the form of a histogram. Data are representative of 4 independent experiments with similar results (*right*). ***P < 0.001 versus vehicle-treated cells

First, we examined whether A77 1726 exerts a positive impact on the Nrf2-mediated HO-1 induction in Jurkat T cells. Nrf2 activity in Jurkat cells treated with A77 1726 was increased in a dose-dependent manner compared with vehicle (DMSO)-treated cells (Fig. 1a). As expected, HO-1 activity in Jurkat cells was also increased by A77 1726 treatment in a dose-dependent manner (Fig. 1b). Next, to confirm the induction property of A77 1726 on the Nrf2-HO-1 axis, A77 1726 was treated in IL-6-stimulated mouse primary T cells isolated from normal C57BL/6 mice. The results also showed the same results (Fig. 1c, d). To investigate the effects of A77 1726 under Th17 cell-polarizing conditions, isolated murine CD4^+^ T cells were cultured in the presence of anti-CD3, anti-CD28, TGF-β, IFN-γ, IL-4 and IL-6 with or without A77 1726 for 72 h. The flow cytometry results showed that Th17 cell differentiation is suppressed by A77 1726 in a dose-dependent manner (Fig. 1e).

### A77 1726 inhibits the arthritis severity in a mouse model of RA

**Fig. 2:**
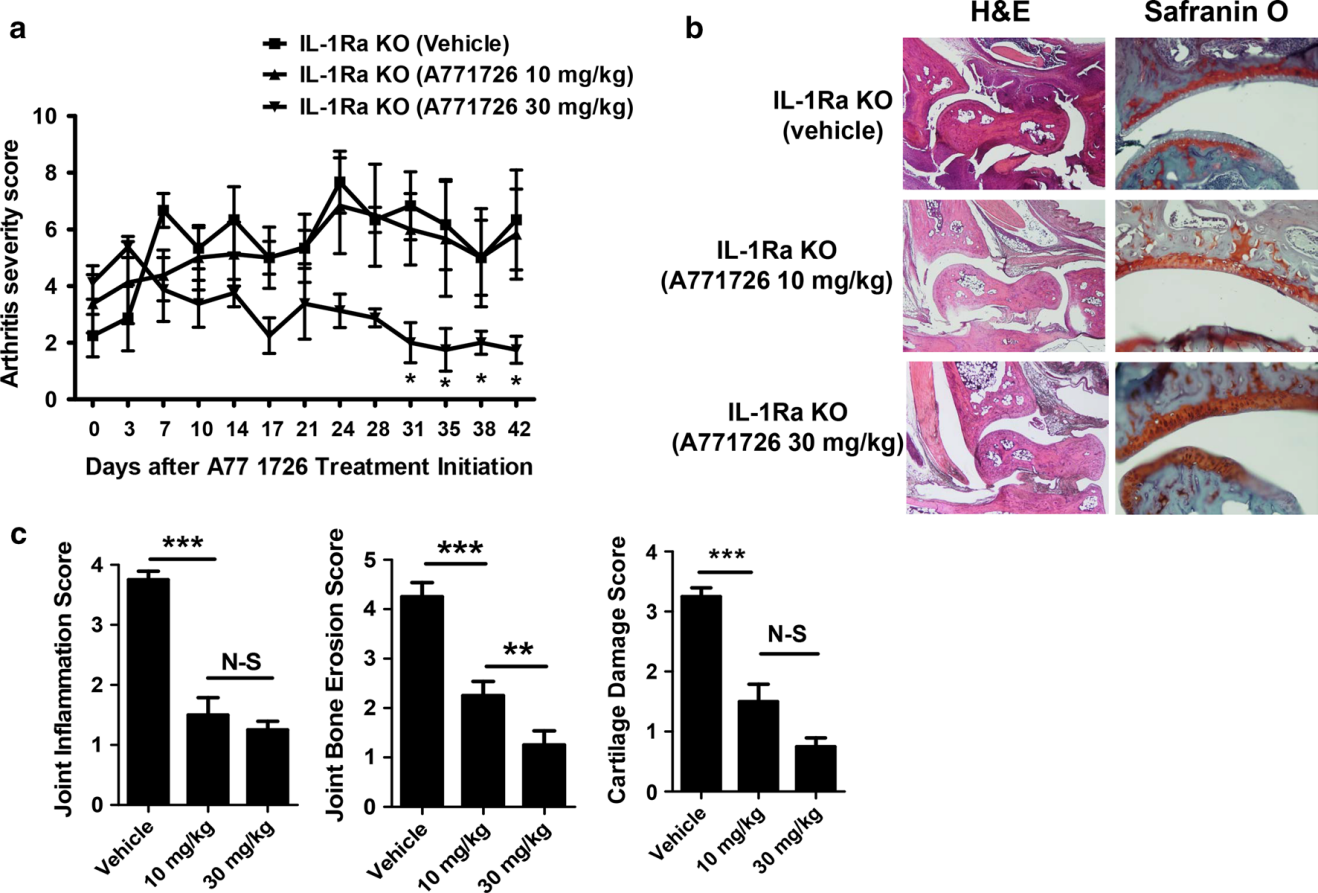
In vivo therapeutic effect of A77 1726 on the development of inflammatory arthritis in mice. **a** Arthritis score and incidence of arthritis in interleukin-1 receptor antagonist-knockout (IL-1Ra-KO) mice following treatment with A77 1726 or vehicle (DMSO). A77 1726 dissolved in DMSO was given intraperitoneally to two different groups (10 or 30 mg/kg; n = 5 mice per group) three times per week for 6 weeks, starting after the first A77 1726 treatment. **b** Representative histological images of joint tissue sections from IL-1Ra-KO mice. Tissue sections from the joints of each mouse on day 42 after the first A771726 or vehicle treatment were stained with hematoxylin and eosin (H&E; original magnification ×40) and Safranin O (original magnification ×200) to examine the severity of arthritis. **c** Histological scores of inflammation, bone erosion and cartilage damage in mice treated with A77 1726 or vehicle (n = 5 mice per group) are shown. Values are the mean ± SD. *P < 0.05; **P < 0.01; ***P < 0.001 versus vehicle-treated mice

Based on the in vitro results above, we investigated whether treatment with A77 1726 would suppress the development and severity of arthritis in vivo. A77 1726 (10 or 30 mg/kg) was administered for 6 weeks to 14–15-week-old male spontaneous arthritic IL-1Ra-KO mice. At the beginning of A77 1726 treatment, the mice of all the groups showed the signs of joints inflammation. Mean arthritis scores at that time were 2.3, 3.4 and 4.1 in vehicle-, 10 mg/kg-, and 30 mg/kg-treated groups, respectively. At the beginning of treatment, there was no significant difference in arthritis severities between the three groups. Treatment of mice with A77 1726 at 30 mg/kg, but not at 10 mg/kg, significantly ameliorated the arthritis severity compared with that in the wild-type and vehicle (DMSO)-treated IL-1Ra-KO mice (Fig. 2a). The clinical severity of arthritis was similar in the mice treated with 10 mg/kg of A77 1726 and the vehicle-treated mice. However, histological sections of the ankle joints stained with H&E and Safranin O showed that arthritis was less severe in A77 1726-treated mice (at both 10 and 30 mg/kg) compared with the vehicle-treated controls (Fig. 2b). There was a statistically significant reduction in the inflammation, bone erosion and cartilage damage scores of the mice treated with 10 or 30 mg/kg A77 1726 compared with those of vehicle-treated arthritis mice (Fig. 2c).

### Anti-inflammatory effects of A77 1726 are associated with reduced expression of inducible nitric oxide synthase (iNOS) and nitrotyrosine in mice with inflammatory arthritis

**Fig. 3:**
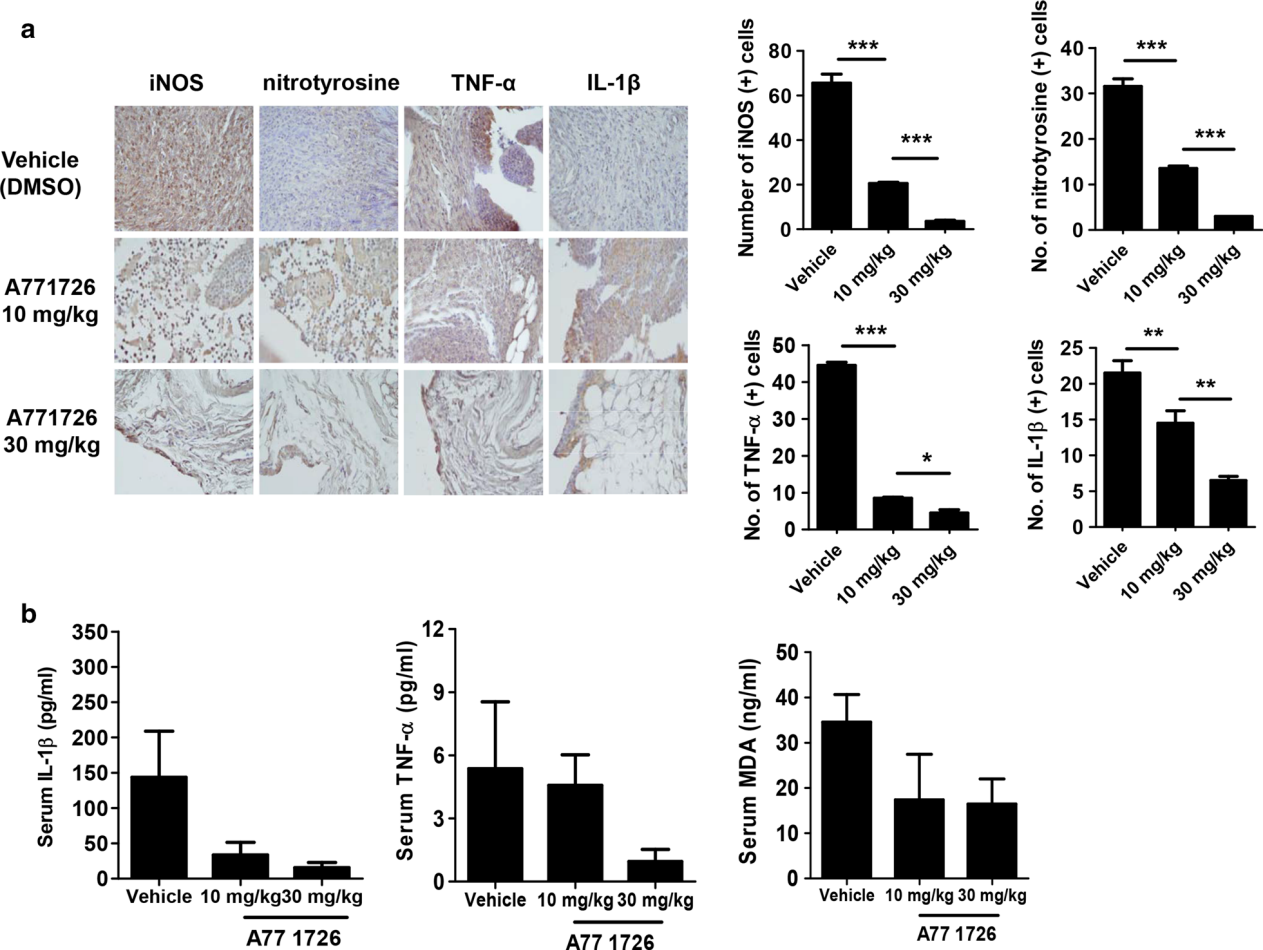
Effects of A77 1726 on inflammatory molecules and oxidative stress in the joints of IL-1Ra-KO mice. **a** Tissue sections from the joints of IL-1Ra-KO mice treated with A77 1726 or vehicle were stained with antibodies to TNF-α, IL-1β, iNOS, or nitrotyrosine. The cells stained with each antibody are shown in* brown* (*left*). Expression of proinflammatory molecules and iNOS and nitrotyrosine (oxidative stress markers) was significantly attenuated in the joints of A77 1726-treated IL-1Ra-KO mice compared with vehicle-treated animals (*right*). **b** Levels of circulating TNF-α, IL-1β and MDA in the serum of IL-1Ra-KO mice in each group. Values are shown as mean ± SD. *P < 0.05; **P < 0.01; ***P < 0.001

We investigated the expression of inflammatory cytokines in vehicle (DMSO)- or A77 1726-treated IL-1Ra-KO mic. TNF-α, and IL-1β are representative proinflammatory cytokines that participate in the inflammatory process in the rheumatoid synovium and have systemic effects [22, 23]. Compared with vehicle-treated IL-1Ra-KO mice, the joints from A77 7126-treated IL-1Ra-KO mice (10 and 30 mg/kg) showed markedly fewer TNF-α- and IL-1β-expressing cells (Fig. 3a). To determine the degree of oxidative stress in the joints, immunohistochemistry was used. The results showed that the expression of nitrotyrosine and iNOS was reduced in a dose-dependent manner in the joints of A77 1726-treated IL-1Ra-KO mice (Fig. 3a). Next, we used ELISA to measure the serum levels of TNF-α, IL-1β, and MDA in the different groups of mice. Although not statistically significant, the concentrations of TNF-α, IL-1β, and MDA tended to be lower in A77 1726-treated arthritis mice than in vehicle (DMSO)-treated animals (Fig. 3b). These results suggest that systemic administration of A77 1726 can inhibit joint inflammation, perhaps via inhibiting oxidative stress.

### A77 1726 treatment decreased the Th17 population and reciprocally increased Foxp3^+^ Treg cell population via regulation of their transcriptional factors

**Fig. 4:**
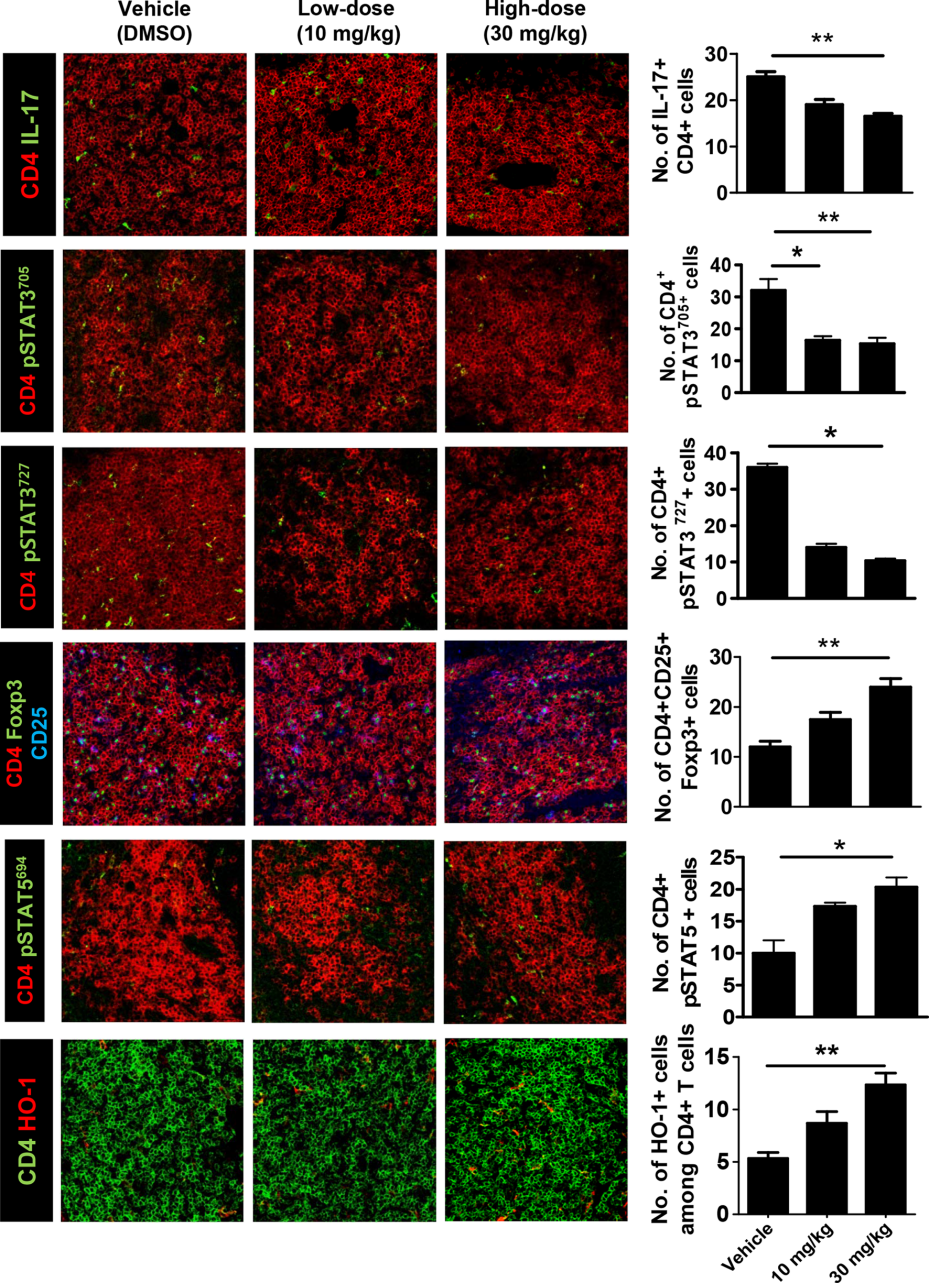
A77 1726 reciprocally regulates Th17/Treg imbalance in vivo. Spleen tissues isolated from each group of mice on day 42 after the first A77 1726 injection were stained for CD4^+^ IL-17^+^ (Th17) cells and CD4^+^ CD25^+^ Foxp3^+^ (Treg) cells using monoclonal antibodies against CD4 (*red*) and IL-17 (*green*) or against CD4 (*red*) CD25 (*blue*), and FoxP3 (*green*). To determine the effects of A77 1726 on the phosphorylation of STAT3 and STAT5 in vivo, isolated spleen tissues was subjected to immunofluorescence staining with monoclonal antibodies against CD4 (*red*), pSTAT3^Tyr705^ (*green*), pSTAT3^Ser727^ (*green*), or pSTAT5^Tyr694^ (*green*). To identify HO-1-expressing CD4^+^ T cells, spleen tissue of IL-1Ra-KO mice with or without A77 1726 treatment was stained with antibodies against CD4 (*green*) and HO-1 (*red*). Original magnification ×400 (*left*). CD4^+^ T cells positive for IL-17, CD25^+^ Foxp3^+^, pSTAT3^Tyr705^, pSTAT3^Ser727^, pSTAT5^Tyr694^, and HO-1 were enumerated visually at higher magnification, as projected on a screen (with each confocal image representative of 4 fields of view), with results quantified as the mean ± SD number of positive cells in 5 mice per group (*right*). *P < 0.05; **P < 0.01

As imbalance between Th17 and Treg cells has been identified as playing a crucial role in RA pathogenesis [24], we investigated whether the populations of Th17 and Treg cells were altered in A77 1726-treated IL-1Ra-KO mice, IL-17-expressing (mainly Th17) and CD25^+^ Foxp3^+^ (mainly Treg) CD4^+^ T cells were analyzed by confocal microscopy. The results demonstrated that spleen tissues from arthritis mice treated with A77 1726 showed dose-dependent increases in the number of Foxp3^+^ Treg cells and reciprocal decreases in the number of Th17 cells compared with spleen tissues from mice treated with vehicle (Fig. 4). To investigate the mechanisms mediating A77 1726-induced reciprocal regulation of Th17 and Treg cell population, the phosphorylated forms of STAT3 and STAT5 were analyzed. A77 1726 treatment in arthritis mice exhibited attenuated expressions of STAT3 activity (both pSTATTyr705 and pSTAT3Ser727) in CD4^+^ T cells, whereas pSTAT5 activity in those cells was significantly augmented (Fig. 4). We conclude that A77 1726 treatment in mice with inflammatory arthritis exerted anti-inflammatory effects through regulating Th17 and Treg cells via modulating their key transcriptional factors. Interestingly, confocal immunostaining in spleens isolated from each group of mice demonstrated the significantly increased population of HO-1-expressing cells among CD4^+^ T cells.

### Antiarthritis effects of A77 1726 are achieved by selective induction of HO-1 in CD4^+^ T cells

**Fig. 5:**
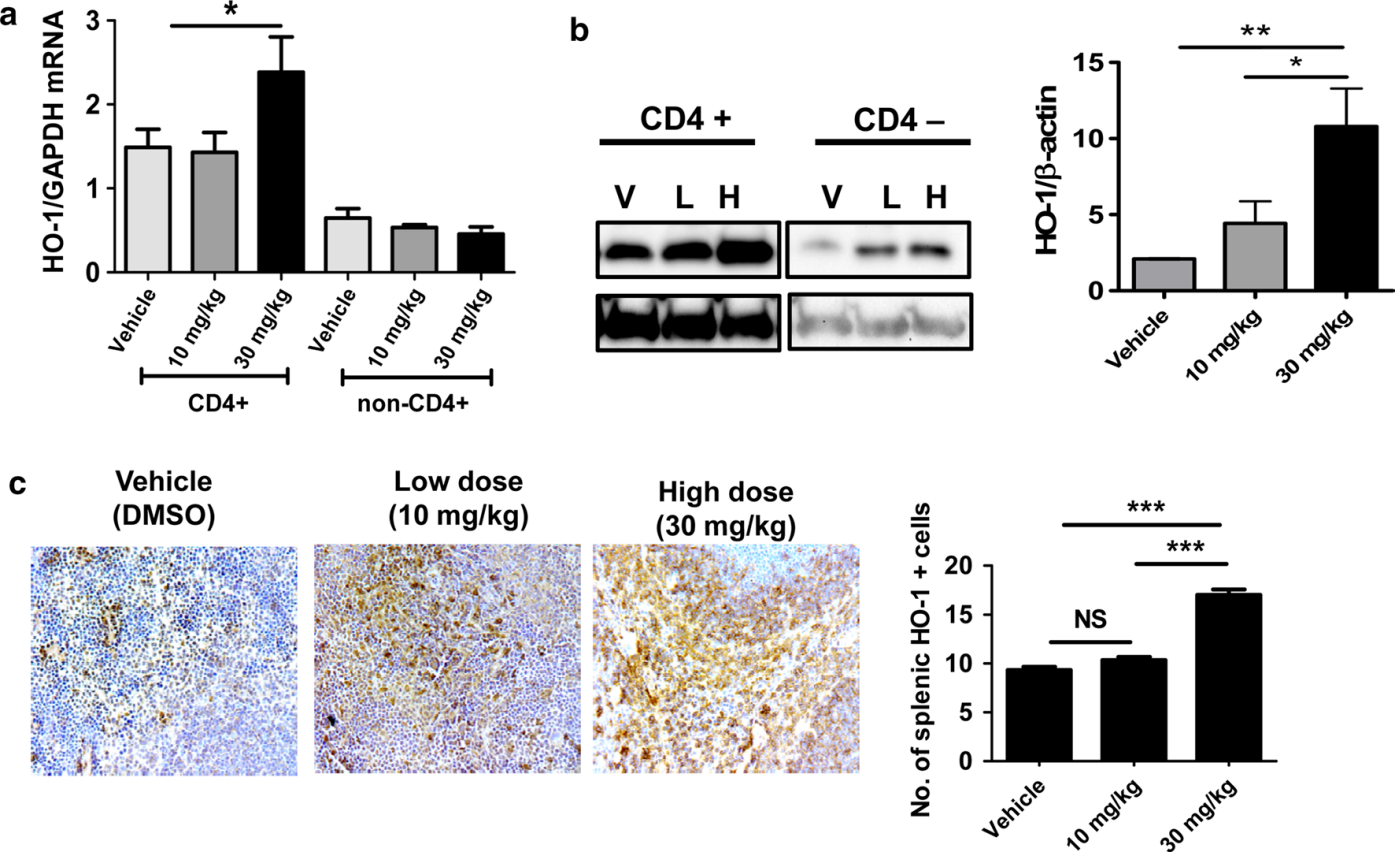
Selectively increased expression of HO-1 in splenic CD4^+^ T cells. **a** mRNA expression of HO-1 in splenic CD4^+^ T cells and non-CD4^+^ splenocytes in each group of mice (n = 5/each group). Values are shown mean ± SD. *P < 0.05. **b** Mouse splenic CD4^+^ T cells and non-CD4^+^ splenocytes were isolated from each group of mice. Lysates were prepared and analyzed by western blotting to detect HO-1. HO-1 expression was higher in a dose-dependent manner in splenic CD4^+^ T cells from A77 1726-treated mice compared with cells from vehicle-treated mice. **c** Representative images of HO-1-positive cells (stained in brown; original magnification ×400) are shown (*left*). The results are quantified as the mean ± SD for the number of HO-1-positive cells (n = 3 mice per group (*right*). *P < 0.05; **P < 0.01; ***P < 0.001

To identify the mechanisms underlying the anti-inflammatory and oxidative stress-reducing properties of A77 1726 and its potential target cells, we analyzed the mRNA expression of ex vivo splenic CD4^+^ T cells and splenic non-CD4^+^ T cells isolated from each group of mice. mRNA expression of HO-1 was significantly increased in splenic CD4^+^ T cells isolated from A77 1726-treated IL-1Ra-KO mice (30 mg/kg) (Fig. 5a). Interestingly, the HO-1-inducing property of A77 1726 was seen only in CD4^+^ T cells and not in non-CD4^+^ splenocytes (Fig. 5a). Next, western blot analysis was conducted to confirm the HO-1 induction effects of A77 1726. The results showed that HO-1 expression in CD4^+^ T cells of arthritis mice was significantly induced by A77 1726 treatment in a dose-dependent manner (Fig. 5b). Immunohistochemical analysis also showed that A77 1726 increased the number of HO-1-expressing splenocytes (Fig. 5c). Taken together, these results suggest that the in vivo anti-inflammatory and oxidative stress-reducing effects of A77 1726 were related to the HO-1-inducing property and that the beneficial effects were restricted to T cells.

## Discussion

The present study showed that the antiarthritis effects of A77 1726 in vivo may be associated with significant expansion of Foxp3-expressing Treg cells and reciprocal suppression of Th17 cell differentiation. And, anti-inflammatory effects of A77 1726 shown in arthritis mice are associated with inhibited oxidative damage and selective HO-1 induction in CD4^+^ T cells. To our knowledge, our study is the first to show an HO-1-inducing activity of leflunomide or its metabolite, A77 1726. Our results suggest the potential use of leflunomide in treating other inflammatory and degenerative diseases by altering the pathogenesis in which impaired HO-1 activity is implicated. From this viewpoint, it may be valuable to understand further the mechanism of action of leflunomide, although the drug is already widely used in RA patients.

HO is a rate-limiting enzyme in heme catabolism and cleaves heme to form biliverdin Ixα, carbon monoxide, and iron [25]. There are two functional isoenzymes of HO, HO-1 and HO-2 [26]. HO-1 represents a stress-responsive protein that is induced by exogenous and endogenous factors such as oxidative stress and inflammatory mediators [27]. By contrast, HO-2 is constitutively expressed and the expression level of HO-2 mRNA is maintained within a narrow range in human cells [28]. HO-1 deficiency in mice is characterized by hepatosplenomegaly, lymphadenopathy, and fibrosis [29]. The genetic absence of HO-1 results in abnormal T cell function and a subsequent proinflammatory condition [29]. Kobayashi et al. [30] demonstrated that synovial tissue from RA patients showed greater expression of HO-1 compared with tissue from people with osteoarthritis or noninflammatory joint diseases. In our study, HO-1 expression was lower in the joint tissues from A77 1726-treated mice compared with control arthritis mice (data not shown). Kobayashi et al. [30] asserted that HO-1 expression may be increased to maintain homeostasis in the inflammatory state of the rheumatoid joint. They found that specific inhibition of HO-1 activity in RA-derived synovial cells resulted in marked increase in inflammatory responses such as TNF-α, IL-6, and IL-8, which suggests that endogenously expressed HO-1 plays a regulatory role in the development of synovial inflammation. Kobayashi et al. suggested that modulation of HO-1 expression may be a novel treatment strategy in human RA.

Leflunomide is classically considered to exert anti-inflammatory and antiarthritis effects through the inhibition of de novo pyrimidine synthesis and therefore to have an antiproliferative effect. The concentrations of A77 1726 used in our in vitro study did not alter the proliferation of Jurkat and mouse primary T cells, but A77 1726 at a concentration of 200 μM exerted antiproliferative effects in the cells (data not shown). These results indicate that lower doses of A77 1726 (up to 100 μM) increase the HO-1 activity while maintaining T cell proliferation, which implies newly identified mechanism of action for leflunomide.

Given the physiological role of HO-1 in protecting against inflammation, some previous studies have suggested a potential role of HO-1 in human RA. Kirino et al. [31] demonstrated that TNF-α, the pivotal cytokine that has a pathophysiological role in RA pathogenesis, suppresses HO-1 expression in RA peripheral blood mononuclear cells. One case–control study including 736 RA patients and 846 healthy controls identified that *HO*-*1* promotor polymorphism is associated with RA susceptibility, which implies that impaired HO-1 activity can induce the development of human RA [32]. An HO-1-inducing strategy may be beneficial in RA patients and in people who are susceptible to the disease.

## Conclusion

In conclusion, systemic administration of A77 1726, the active metabolite of leflunomide, reduced clinical arthritis severity and histological inflammation in this mouse model of RA. A77 1726 treatment significantly inhibited oxidative damage and reduced proinflammatory cytokine expression in inflamed joints. The antiarthritis effect of A77 1726 may be associated with significant induction of HO-1 activity in CD4^+^ T cells and reciprocal regulation of Th17^−^ Treg cells balance. These data suggest that an HO-1-inducing strategy may be a new therapeutic target in RA patients.

## Abbreviations

APC: allophycocyanin
BSA: bovine serum albumin
DMARD: disease-modifying antirheumatic drug
DMSO: dimethyl sulfoxide
EDTA: ethylenediaminetetraacetic acid
ELISA: enzyme-linked immunosorbent assay
H&E: hematoxylin and eosin
HO-1: heme oxygenase-1
IFN-γ: interferon-γ
IL-1β: interleukin 1β
IL-1Ra-KO: interleukin-1 receptor antagonist-knockout
iNOS: inducible nitric oxide synthase
MDA: malondialdehyde
Nrf2: nuclear-related factor 2
PBS: phosphate-buffered saline
PCR: polymerase chain reaction
PE: phycoerythrin
RA: rheumatoid arthritis
TGF β: transforming growth factor β
Th17: IL-17-producing CD4^+^ T
TNF-α: tumor necrosis factor-α
Treg: regulatory T
